# Additive effects of EEG neurofeedback on medications for ADHD: a systematic review and meta-analysis

**DOI:** 10.1038/s41598-022-23015-0

**Published:** 2022-11-27

**Authors:** Feng-Li Lin, Cheuk-Kwan Sun, Yu-Shian Cheng, Ming Yu Wang, Weilun Chung, Ruu‐Fen Tzang, Hsien‐Jane Chiu, Ying-Chih Cheng, Kun-Yu Tu

**Affiliations:** 1 Taoyuan Psychiatric Center, Ministry of Health and Welfare, Taoyuan City, Taiwan; 2 Department of Emergency Medicine, E-Da Hospital, I-Shou University, Kaohsiung, Taiwan; 3 School of Medicine for International Students, College of Medicine, I-Shou University, Kaohsiung, Taiwan; 4 Department of Psychiatry, Tsyr-Huey Mental Hospital, Kaohsiung Jen-Ai’s Home, Kaohsiung, Taiwan; 5 Institute of Biomedical Sciences, National Sun Yat-Sen University, Kaohsiung, Taiwan; 6 Department of Psychiatry, China Medical University Hsinchu Hospital, China Medical University, Hsinchu, China; 7 Department of Health Services Administration, China Medical University, Hsinchu, China; 8 Department of Psychiatry, Mackay Memorial Hospital, Taipei, Taiwan; 9 Institute of Hospital and Health Care Administration, National Yang Ming Chiao Tung University, Taipei, Taiwan; 10 Institute of Epidemiology and Preventive Medicine, College of Public Health, National Taiwan University, Taipei, Taiwan; 11 Research Center of Big Data and Meta-Analysis, Wan Fang Hospital, Taipei Medical University, Taipei, Taiwan

**Keywords:** Psychology, Diseases

## Abstract

To elucidate possible additive effects of electroencephalogram-based neurofeedback (EEG-NF) on medications against the core symptoms of attention-deficit/hyperactivity disorder (ADHD), randomized controlled trials (RCTs) were retrieved from electronic databases including PubMed, EMBASE, ClinicalKey, Cochrane CENTRAL, ScienceDirect, and ClinicalTrials.gov from inception to March 2022. The primary outcomes were changes in ADHD symptoms (i.e., global, inattention, hyperactivity/impulsivity) assessed with validated rating scales, while secondary outcome was all-cause discontinuation rate. Meta-analysis of five RCTs involving 305 participants [Median age = 9.285 years (range 8.6–11.05)] with a median follow-up of 12 weeks showed additive effects of EEG-NF on medications from parents’ observations against ADHD global symptoms (Hedges’ g = 0.2898, 95%CI [0.0238; 0.5557]) and inattention symptoms (Hedges’ g = 0.3274, 95%CI [0.0493; 0.6055]). However, additive effects failed to sustain six months after EEG-NF intervention. Besides, there was no difference in improvement of hyperactivity/impulsivity from parents’ observation, attentional performance, and all-cause discontinuation rate between the two groups. Our results supported additional benefits of combining EEG-NF with medications compared to medication alone in treating global symptoms and symptoms of inattention in ADHD patients. Nevertheless, given a lack of evidence showing a correlation between underlying physiological changes and small effect sizes in our preliminary results, further studies are warranted to support our findings.

## Introduction

Although attention-deficit/hyperactivity disorder (ADHD) mainly comprises symptoms of inattention and hyperactivity, it is also associated with many other behavioral and emotional issues that contribute to a variety of psychosocial impairments^1^. While causes of ADHD may be heterogeneous^1^, ADHD is believed to be a lifespan neurodevelopmental condition with onset in childhood according to the criteria described in Diagnostic and Statistical Manual of Mental Disorders, Fifth Edition (DSM-5)^2^. Because children with ADHD are likely to suffer from continuous problems related to their difficulties in attention or impulse controls from an early stage of development, early training and interventions are critical^3^ taking into account the relatively high neuroplasticity of the brain at a young age^4^.

The established interventions for school-aged children with ADHD include both pharmacological and non-pharmacological interventions^1^. Although psychostimulants such as methylphenidate (MPH) have been shown to be highly effective against the core symptoms of ADHD in previous randomized controlled trials (RCTs)^5^, large-scale network meta-analysis^6^, and umbrella review^7^, non-pharmacological interventions including cognitive training, social skill training, behavioral therapies, and biofeedback may provide additional benefits in the treatment of other associated behavioral problems^5,7^. Moreover, the side-effects associated with pharmacological treatments remain important concerns of caregivers^8^. However, the results of previous reviews and meta-analyses showed that psychosocial interventions such as cognitive training seemed less effective than pharmacological approaches^7^. Therefore, another new non-pharmacological therapeutic strategy through device-assisted biofeedback that targets self-regulation of underlying abnormal brain pathophysiology, such as electroencephalogram-based neurofeedback (EEG-NF), has become an important area of research focusing on its potential as an alternative treatment approach to ADHD^9^.

The rationale of EEG-NF originated from the observation of certain brain-wave patterns associated with the symptoms of ADHD^10^, in particular an increase in theta/beta ratio^11^. Therefore, by targeting these abnormal brain wave patterns through real-time feedback from electroencephalography, patients diagnosed with ADHD were trained to correct these EEG abnormalities via operant conditioning^10^. The most commonly used protocol for EEG-NF is theta/beta ratio (TBR) which aims at decreasing theta and/or increasing beta power^10^. Another standard protocol, which was developed later, targets the slow-cortical potential (SCP) and involves self-regulation of cortical activation and inhibition^12^. Although there have been a number of clinical trials investigating the efficacy of EEG-NF on ADHD symptoms over the past two decades^13^, the clinical efficacy and specificity of EEG-NF effects remain unclear^9^. For example, despite the reported effectiveness of EEG-NF against the symptoms of ADHD with a sustainable long-term effect in previous meta-analyses^13,14^, those meta-analyses did not demonstrate a superior effectiveness of EEG-NF compared to that of active control conditions such as medications, cognitive training or behavioral therapies^13,14^. A recent meta-analytical investigation also demonstrated superior therapeutic effects of pharmacological interventions in comparison with EEG-NF^15^. Several studies further found that specific neurophysiological effects, such as a specific increase in P3 amplitude, was only observed in the medication treatment group but not in the EEG NF group, suggesting that medications may produce more specific therapeutic effects on the underlying brain pathophysiologies^16,17^. Nevertheless, medications and EEG-NF may benefit patients with ADHD through different mechanisms^5,15^, while the former directly targets brain neurotransmitters^18^, improvement in ADHD symptoms through EEG-NF is based on self-regulation through operant conditioning^19^. Several RCTs also found that EEG-NF in combination with medications were superior to medication treatments alone^20,21^. Hence, non-pharmacological interventions such as EEG-NF may offer additional benefit to existing pharmacological treatment rather than being viewed as a single treatment modality.

Although there were several previous meta-analyses focusing on the therapeutic effectiveness of EEG-NF on the symptoms of ADHD^13–15,22,23^, none of them focused on the efficacy of combining medications and EEG-NF. Therefore, the present meta-analysis aimed at testing the hypothesis of possible additive therapeutic effects of EEG-NF on medications for symptoms of ADHD. We also explored the sustainability of the benefits of EEG-NF after NF interventions.

## Methods and materials

### Data sources and search strategy

This systematic review and meta-analysis followed the PRISMA statement guidelines^24^. Literature was searched on electronic databases including PubMed, EMBASE, ClinicalKey, the Cochrane CENTRAL, ScienceDirect, and ClinicalTrials.gov from the earliest available date to March 2022. The protocol for this study was registered in PROSPERO systematic review protocol registry (No. CRD42022339341).

The literature search was conducted by three independent researchers (YC Cheng, YS Cheng, and CK Sun) with the following key terms: “(neurofeedback) AND (attention or attention-deficit/hyperactivity disorder or ADHD)”. The keywords and limits used for each database are provided in eTable 1. RCTs investigating the effect of combination therapies of neurofeedback and medication for ADHD were eligible for review. Additional eligible studies were identified by searching the reference lists of the initially retrieved articles and relevant reviews^13–15^.

### Inclusion and exclusion criteria

We aimed at investigating the additive effects of EEG neurofeedback on medications against the symptoms of ADHD. Eligible studies included: (1) human randomized controlled trials; (2) patients who were given a clinical diagnosis of ADHD; (3) clinical trials that compared the therapeutic efficacy of combined therapies of EEG-NF and medications with medication alone. We excluded studies that used control conditions other than medications for the treatment of ADHD, those that were not RCT, and animal experiments.

### Data extraction and quality assessment

Three investigators (YC Cheng, YS Cheng, and CK Sun) independently extracted relevant information from the included studies and evaluated the methodological quality by using the Cochrane Collaboration risk of bias tools. The following data were obtained from the studies including the last name of the first author, publication year, total sample size, number of study participants in intervention and control groups, gender prevalence, intelligent quotient (IQ), age characteristics, and types of medications being used. The Cochrane Collaboration’s tool was used to evaluate seven domains of risk: selection bias (sequence generation and concealment), performance bias (blinding of participants and assessors), detection bias (blinding of outcome assessment), attrition bias (incomplete outcome data), selective outcome reporting, and other bias. Each item was classified as low, high, or an unclear risk of bias (if there was insufficient information). Disagreements among the three investigators were resolved through discussion.

### Efficacy outcomes

The main outcomes were changes in symptoms of ADHD, namely global, inattention, hyperactivity, and impulsivity, assessed with any clinically validated rating scale or changes in test scores for attentional performance such as continuous performance test (CPT), after interventions. The secondary outcome was acceptability (defined as all-cause discontinuation measured by the proportion of patients who withdrew from the study for any reason). We also conducted a subgroup analysis to investigate the sustainability of treatment efficacies after NF intervention.

The means and standard deviations of changes from baseline were extracted. The authors of studies with missing data were contacted by emails in an attempt to retrieve the necessary information. All potentially relevant manuscripts were independently reviewed by two investigators (YC Cheng and YS Cheng) and areas of disagreement or uncertainty were adjudicated by a third investigator (CK Sun).

### Statistical analysis

To assess the overall effect size, we calculated the standardized mean differences (SMDs) using the formula for Hedges’ g with 95% confidence intervals for continuous outcomes. For dichotomous outcomes, we computed the odds ratio (OR). SMD was used to compare the change in scores after intervention between the intervention and control groups. When measurement was reported in multiple time points, we only extracted the data from baseline and the final time points. SMD was calculated from the mean and SD of the scores. A positive effect size indicated superior effects of the intervention versus the control groups. The SDs of the changes in scores from baseline were calculated using the formula {SD = square root [(SD pre-treatment)^2^ + (SD post-treatment)^2^ − (2R × SD pre-treatment × SD post-treatment)], assuming a correlation coefficient (R) = 0.5} if they were not available in the studies. When only the standard error of the mean (SEM) was reported, standard deviation (SD) was calculated by multiplying the SEM by the square root of the sample size. For studies using median and range, mean and SD were estimated using the formula according to the Cochrane guidelines^25,26^. For adverse effects expressed as binary outcomes, the effect size was similarly calculated using the odds ratio (OR).

The possible sources of heterogeneity or inconsistency among trials were investigated. Heterogeneity was assessed using the *I*^2^ test^27^. A random effects model was used on the assumption that the true effect size could vary among studies to offer more generalizable results. Publication bias was evaluated by examining asymmetry in a funnel plot that depicted the effect size against the standard error. Egger’s regression test was also used to assess publication bias when there were 10 or more datasets^28^. Leave-one-study-out sensitivity analysis was performed by sequential exclusion of each trial at a time to examine whether the pooled effects remained robust. To explore the potential effect of trial level modifiers, we considered several covariates (i.e., mean age, total number of EEG-NF sessions, female proportion, treatment duration and IQ) in the meta-regression approaches. However, meta-regression would only be considered if there are more than ten trials that reported on specific outcomes in a meta-analysis as recommended in the Cochrane Handbook for Systematic Reviews of Interventions to provide reliable results^29^. All meta-analytic computations were performed with the R software (R × 64 version 4.1.2, The Cochrane Collaboration, Oxford, UK).

## Results

### Baseline characteristics of included studies

**Figure 1:**
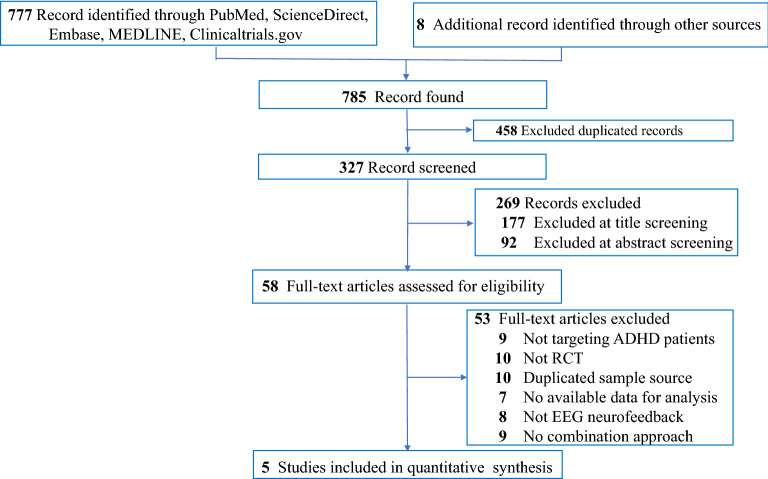
PRISMA diagram of identifying eligible studies.

**Figure 2:**
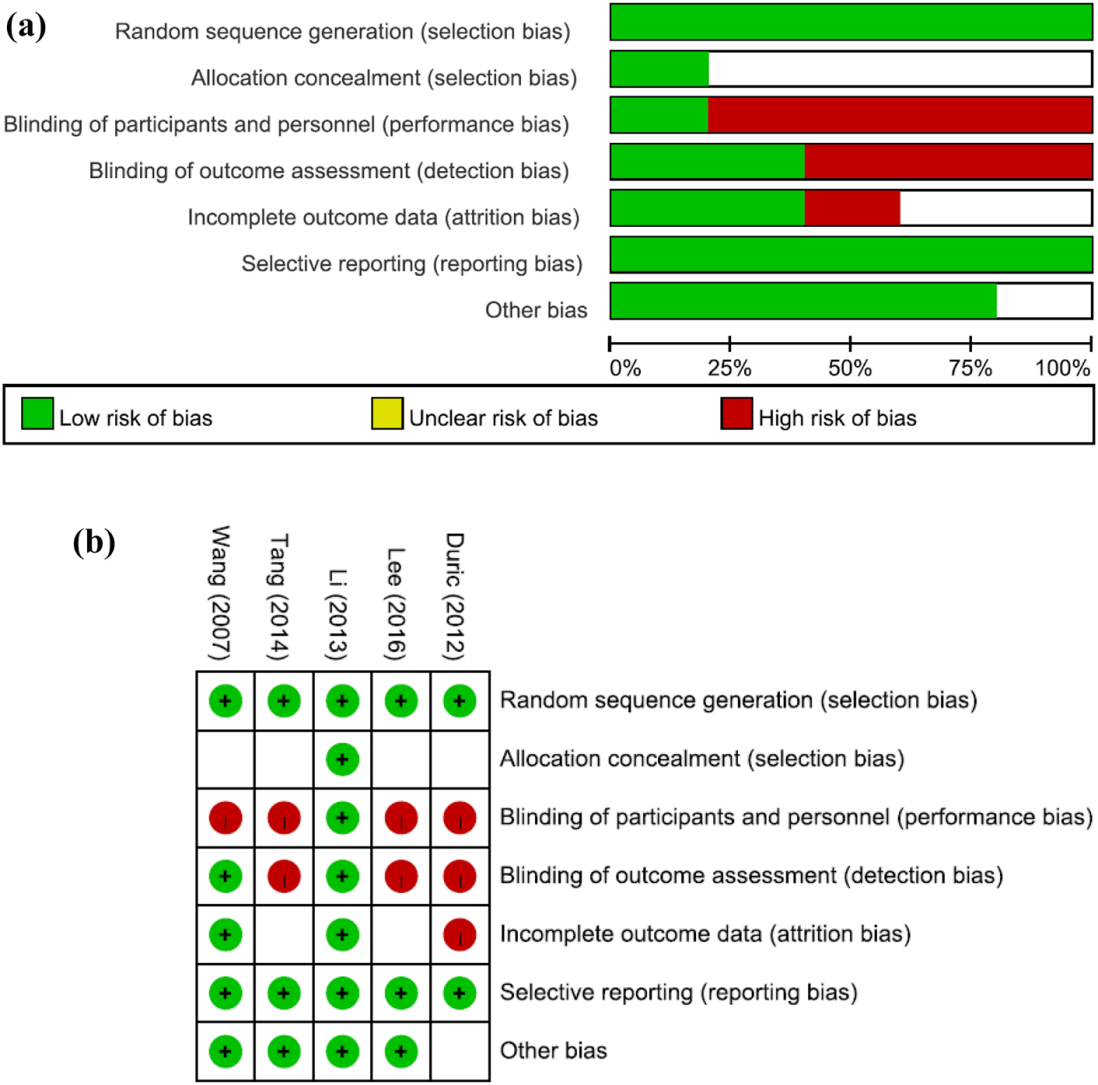
(**a**) Risk of bias summary (**b**) Risks of bias of the included studies.

Figure 1 summarizes the review flowchart in accordance with the PRISMA statement^30^. Of the 327 original studies screened, five RCTs involving a total of 305 participants met the inclusion criteria^20,21,31–33^. Reasons for study exclusions are provided in eTable 2. A summary of the included studies is presented in Table 1. The sample size ranged from 36 to 80. The median age of the participants was 9.285 (range: 8.6–11.05 years) with a median follow-up period being 12 weeks (range: 8–20 weeks). The median number of neurofeedback treatment sessions was 30 (range: 16–40 weeks). All included trials used TBR protocols for their EEG-NF. Four studies used MPH^21,31–33^ while the other trial did not specify the type of medications used for ADHD^20^. Details about the dose strategy of MPH are provided in Table 1. The results of quality assessment of the included trials using the Cochrane Collaboration tool based on authors’ judgments regarding the risk of bias in each item are presented in Fig. 2a and b. Overall, since only one of all the included trials used a double-blind design^21^, the performance and detection biases were the main source of biases in most studies.

**Table 1:** Summary of characteristics of included studies in the current meta-analysis.

Author (year) [Ref]	Criteria	Comparison	Session	N	Duration (weeks)	Outcome	Subtype	IQ	Age (years)	Female (%)	Country
Lee (2017)^20^	DSM-IV-TR	EEG-NF (TBR) + Medication	20	18	10	1.ADHD RS: total, inattention, and H/I 2.ADHD Diagnostic System	Inattention (41.67%) Combined (44.44%) H/I (13.89%)	100.39	8.75 (6–12 years)	25	Korea
		Medications		18							
Tang (2014)^31^	DSM-IV	EEG-NF (TBR) + MPH: 18–54 mg/d	16	32	8	1.Chinese ADHD rating scale: total, inattention, and H/I 2.Attentional test: d2 test	N/A	N/A	8.6 (7–12 years)	45.31	China
		MPH		32							
Li (2013)^21^	DSM-IV	EEG-NF (TBR) + MPH: optimal dose (starting 5–10 mg/d, max. 60 mg/day)	40	32	N/A	ADHD RS-IV: total, inattention, and H/I	Inattention (65.6%) Combined (29.7%) H/I (4.7%)	N/A	10.6 (7–16 years)	15.6	China
		MPH		32							
Duric (2017)^33^	ICD-10	EEG-NF (TBR) + MPH 1 mg/kg/d	30	30	12	Barkley’s Defiant Children: total, inattention, and H/I	N/A	86.02	11.05 (6–18 years)	18.03	Norway
		MPH		31							
Wang (2007)^32^	DSM-IV	EEG-NF (TBR) + MPH optimal dose (starting 5 mg/d, max. 30 mg/day)	40	40	20	1.Chinese Conner's behavioral rating scale: total, inattention, and H/I 2.Integrated visual and auditory continuous performance test	Inattention (58.75%) Combined (32.5%) H/I (8.75%)	93.685	9.285 (6–16 years)	15	China
		MPH		40							

*ADHD-RS* ADHD Rating Scale, *ADHD-RS-IV* ADHD Rating Scale-IV, *d* day, *DSM-IV* diagnostic and statistical manual of mental disorders, fourth edition, *DSM-IV-TR* diagnostic and statistical manual of mental disorders, fourth edition, text revision, *EEG-NF* electroencephalographic biofeedback, *H/I* hyperactivity/impulsivity, *ICD-10* International Classification of Diseases, 10th Revision, *IQ* Intelligence Quotient, *MPH* methylphenidate, *N* number, *N/A* not available, *TBR* theta/beta ratio.

### Pooled effects of neurofeedback on ADHD symptoms

**Figure 3:**
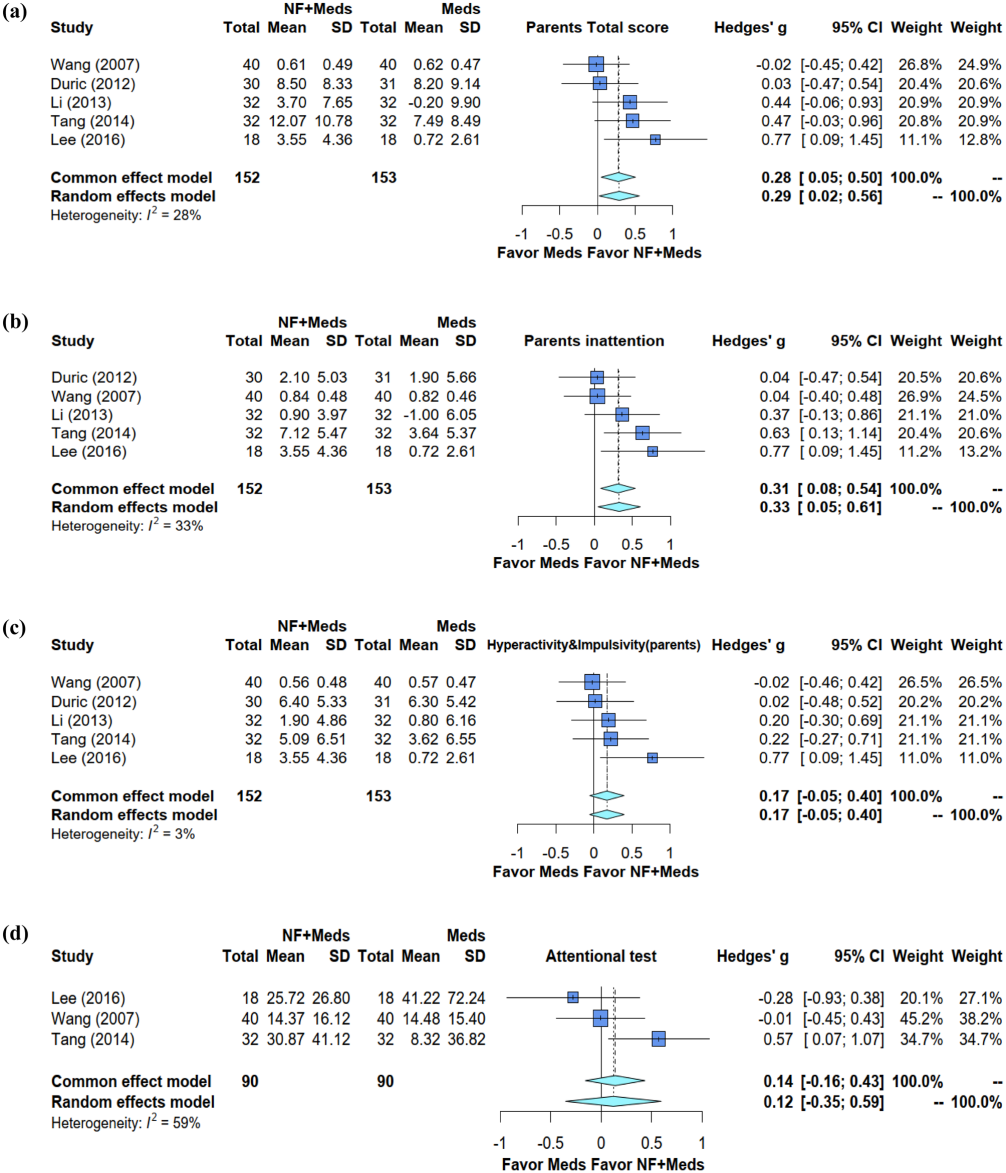
Forest plot of effect sizes for comparing the difference between combined treatment (EEG-NF + medications) and medication alone groups (**a**) in the improvement of global symptoms (**b**) inattention, (**c**) hyperactivity/impulsivity, and (**d**) attentional performance.

**Figure 4:**
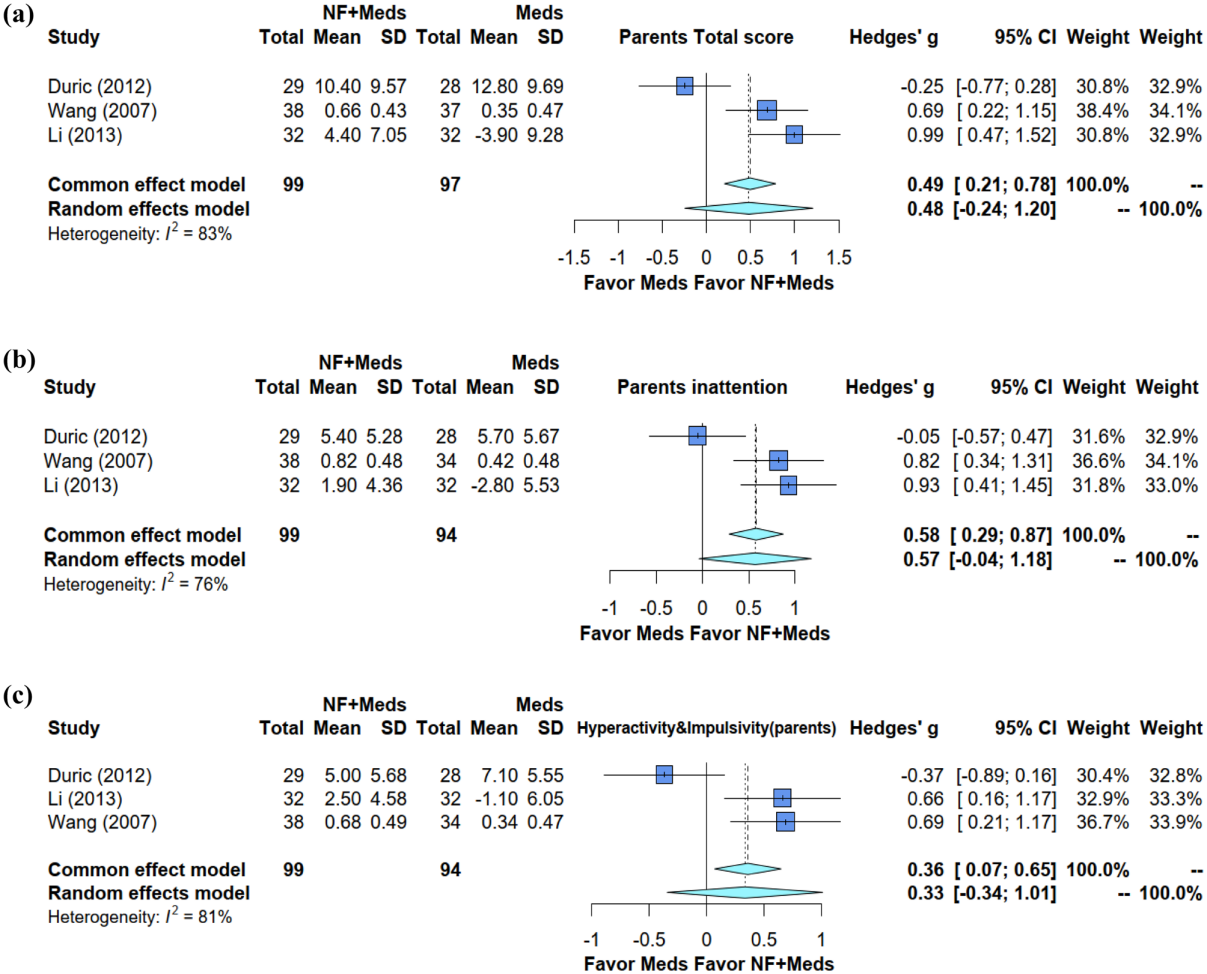
Forest plot of effect sizes for comparing the difference between combined treatment and medication alone groups 6 months after NF intervention (**a**) in the improvement of global symptoms (**b**) inattention, and (**c**) hyperactivity/impulsivity,

The results of each meta-analysis are summarized in Table 2. The meta-analysis of five trials involving 305 participants showed an additive beneficial effect of EEG-NF on medications against the global symptoms of ADHD from parents’ observation (Hedges’ g = 0.2898, 95% CI [0.0238; 0.5557], *p* = 0.0327, *I*^2^ = 28.3%) (Fig. 3a). However, the additive effect of neurofeedback on medications lost its statistical significance six months after NF intervention (Hedges’ g = 0.4807, 95% CI [− 0.2430; 1.2044], *p* = 0.1930, *I*^2^ = 83.2%) (Fig. 4a). For symptoms of inattention from parents’ observation, our results also showed an additive beneficial effect of EEG-NF on medications (Hedges’ g = 0.3274, 95% CI [0.0493; 0.6055], *p* = 0.0210, *I*^2^ = 33.1%) (Fig. 3b). Similar to that in the treatment of global symptoms, the additive effect of neurofeedback on medications for symptoms of inattention lost its significance six months after NF intervention despite a trend of better effect for the combined approach compared with medication alone (Hedges’ g = 0.5706, 95% CI [− 0.0370; 1.1783], *p* = 0.0657, *I*^2^ =76.4%) (Fig. 4b). Regarding the symptoms of hyperactivity/impulsivity from parents’ observation, the combined approach was not superior to medication alone in its therapeutic effects (Hedges’ g = 0.1714, 95% CI [− 0.0544; 0.3971], *p* = 0.1368, *I*^2^ =3.1%) (Fig. 3c) immediately or six months after NF intervention (Hedges’ g = 0.3336, 95% CI [− 0.3438; 1.0110], *p* = 0.3344, *I*^2^ = 81.2%) (Fig. 4c). Focusing on the improvement in test scores for attentional performance, combining EEG-NF with medications offered no additional therapeutic benefit compared to medication alone (Hedges’ g = 0.1201, 95% CI [− 0.3531; 0.5933], *p* = 0.6189, *I*^2^ =58.6%) (Fig. 3d). Visual inspection of the funnel plot did not reveal asymmetry for any of the above results, suggesting a low risk of publication bias.

**Table 2:** The comparison of improvements in symptoms of ADHD between EEG-NF + Medications vs Medication alone from parents’ observation.

	No. of studies	Patients/controls	Effect sizes (95% CI)	Effect size p value	Heterogeneity *I*^2^ (%)
Global symptoms	5	152/153	0.2898 (0.0238; 0.5557)	0.0327*	28.3%
Global symptoms (Follow up)	3	99/97	0.4807 (−0.2430; 1.2044)	0.1930	83.2%
Inattention	5	152/153	0.3274 (0.0493; 0.6055)	0.0210*	33.1%
Inattention (Follow up)	3	99/94	0.5706 (−0.0370; 1.1783)	0.0657	76.4%
Hyperactivity/Impulsivity	5	152/153	0.1714 (−0.0544; 0.3971)	0.1368	3.1%
Hyperactivity &Impulsivity (Follow up)	3	99/94	0.3336 (−0.3438; 1.0110)	0.3344	81.2%
Attentional test	3	90/90	0.1201 (−0.3531; 0.5933)	0.6189	58.6%
All-cause discontinuation rate	3	99/94	0.9364 (0.4220; 2.0780)	0.8717	0.0%

There was no significant difference in all-cause discontinuation rate between the combination and control groups (OR 0.9364, 95% CI [0.4220; 2.0780], *p* = 0.8717, *I*^2^ =0.0%).

### Sensitivity analysis

The stability of the results from the current meta-analysis and subgroup analyses was tested through a leave-one-out sensitivity analysis that showed inconsistent outcomes for the analysis of global symptoms, subgroup analysis of global symptoms in follow-up stage, analysis of symptoms of inattention, subgroup analysis of symptoms of inattention in follow-up stage, and subgroup analysis of symptoms of hyperactivity/impulsivity in follow-up stage (eTable 3). Therefore, the robustness of evidence generated from the current investigation regarding the above analyses remains to be elucidated.

## Discussion

Although EEG-NF has been reported to be an effective treatment against the symptoms of ADHD from both raters’ observations^13,23^ and subjective reports^22^, a recent meta-analysis demonstrated an inferior efficacy of EEG-NF in comparison with methylphenidate^15^. On the other hand, combined pharmacological and non-pharmacological interventions may still provide additional benefits for patients with ADHD^5,7,15^. Our meta-analysis was the first to demonstrate an additive effect when EEG-NF was used in conjunction with medications against the global symptoms and the symptoms of inattention for patients with ADHD. Subgroup analysis focusing on sustained therapeutic effects further showed a trend of better but non-significant improvement in inattention six months after NF intervention (ES = 0.57, *p* = 0.065). Nevertheless, the additional benefits of combining EEG-NF with medications for symptoms of ADHD, which were derived from only five RCTs and a relatively small number of total participants (n = 305), should be considered preliminary and warrant further studies for elucidation.

Consistent with the results of previous meta-analyses^13,22,23^, our findings showed a superior effectiveness of a combined treatment for symptoms of inattention compared to that for hyperactivity/impulsivity. In fact, although there was a trend of better improvement in symptoms of hyperactivity/impulsivity in the combined treatment group than in the medication-only group, this difference was not statistically significant. Since all of the included studies adopted theta/beta protocols which focused on enhancing arousal^10^, its therapeutic effect may be better reflected by arousal-related symptoms such as inattention. Our results, therefore, were not representative of other NF protocols such as slow cortical potentials (SCP). Moreover, our findings may be more applicable to selected groups of ADHD patients, because all five trials recruited participants with similar characteristics including an age range of 6–18 years, a predominance of male participants, adoption of only theta/beta protocol, exclusion of participants with intellectual disability, and a very low proportion of patients with the hyperactivity/impulsivity subtype. Taking into account the availability of only five RCTs in the current study, more trials including participants of diverse demographic backgrounds and using different NF protocols are required to further elucidate the treatment effectiveness of combining EEG-NF with medications in actual clinical practice. Moreover, despite current evidence showing an additional benefit of combining EEG-NF with medications, given the high cost of EEG-NF (USD130–225 per treatment for 30–40 sessions)^34^ and the relatively small ES (i.e., 0.29) from the current meta-analysis, the cost-effectiveness of EEG-NF remains an important concern that should be discussed with patients or their guardians.

Focusing on individual studies, we found some discrepancies in ESs for therapeutic effectiveness of the combined approach among the included RCTs despite similar characteristics of their participants. In particular, although four out of the five included studies exclusively used methylphenidate as their pharmacological intervention, one^20^, which also showed the largest ES for therapeutic efficacy of the combined approach, did not specify its medication regimen. Since previous evidence has demonstrated an apparent variation in treatment effectiveness among different medications for ADHD^7^, the lack of information about medications in that study^20^ may introduce bias in favor of EEG-NF use as reflected by the results of our sensitivity analysis. With regard to dosing strategies, one study used a fixed dose of 1 mg/kg/day^33^, two studies adopted the best dose approach (17, 18), and two did not provide relevant information^20,31^. Although previous research suggested that the therapeutic benefits of psychosocial interventions in ADHD patients may be more prominent in those treated with relatively low doses of methylphenidate compared with those under high-dose treatment^35^, we were unable to perform a meta-regression or subgroup analysis to investigate the association between medication dosage and therapeutic effectiveness of the combined approach. Further studies about dosing strategies are required to address this issue. Finally, despite the previous identification of placebo effects, which contributed to both performance and detection biases, as the most important methodological problem in previous RCTs that investigated the therapeutic effects of EEG-NF^13,23,36,37^, only one of our included studies used a sham control^20^. Although that trial showed a better therapeutic effect of the combined approach than medication alone with a small ES, one study is not enough to provide tangible evidence. Taken together, the limited number of RCTs included in the current investigation warrants further studies for exploring the confounding effects of the above issues and also those from other unidentified factors.

Focusing on the sustainability of therapeutic benefits, our subgroup analysis failed to demonstrate a significantly better treatment effect of the combined approach compared with medications six months after intervention. Nevertheless, given the availability of only three RCTs available for subgroup analysis and as well as the moderate effect size regarding the sustained therapeutic effects for inattention (ES: 0.57) with borderline significance (*p* = 0.0657), our result was still in favor of a better sustained effect of the combined approach than medication alone, especially for the symptoms of inattention. Together with a previous meta-analysis showing that EEG-NF may offer a durable therapeutic effect^14^, further studies are required to shed light on the additional long-term benefits of the combined approach.

There are several limitations in the present meta-analysis. First, given the limited number of available RCTs involving a total of only 305 participants, our findings were only preliminary. Second, the lack of blinding in most of the included studies rendered them highly susceptible to performance and detection biases. More studies with a double-blind design are needed to elucidate the benefits of the neurofeedback approach other than those from motivation enhancement^36^. Fourth, limited information about medication dosage precluded our analysis of its potential impacts on the effectiveness of EEG-NF in this clinical setting. Fifth, because none of the included studies reported TBR threshold in their inclusion criteria, we could not rule out the possibility of underestimating the benefits of the TBR protocol by including studies that recruited patients who started with a relatively low TBR (i.e., < 4.5)^38^. Finally, given the relatively similar characteristics of participants in the included trials and the Asian origins in four out of the five studies, our findings may not be extrapolated to populations of different demographic characteristics or ethnicities.

## Conclusion

Our results supported additional beneficial therapeutic effects of combining EEG-NF with medications compared to medication treatment alone against the global symptoms and the symptoms of inattention in patients with ADHD. However, given the limited number of included trials, the inconclusive evidence regarding the sustainability of the therapeutic effects of NF and a lack of evidence showing a correlation between underlying physiological changes and improvements in ADHD symptoms from EEG-NF training, further large-scale randomized controlled trials are warranted to support our findings.

## Supplementary Information

Supplementary Tables.

## Data Availability

The datasets used and/or analyzed during the current study available from the corresponding author on reasonable request.
