# Supplementary material for: Additive effects of EEG neurofeedback on medications for ADHD: a systematic review and meta-analysis
Source: Sci Rep. 2022 Nov 27;12:20401. doi: 10.1038/s41598-022-23015-0 (PMC9701807; doi:10.1038/s41598-022-23015-0)
Supplement: Supplementary file 1 — Supplementary Tables. [file 41598_2022_23015_MOESM1_ESM.docx]

**eTable 1: applied keyword and the search result in each database**

| Database | Keyword | Filter | Date | Result |
| --- | --- | --- | --- | --- |
| PubMed | (neurofeedback) AND (attention or attention-deficit/hyperactivity disorder or ADHD) | RCT | 2022/06/10 | 98 |
| Embase | (neurofeedback) AND (attention or ADHD) | RCT | 2022/06/10 | 74 |
| ClinicalKey | (neurofeedback) AND (attention or attention-deficit/hyperactivity disorder or ADHD) | RCT | 2022/06/10 | 14 |
| Cochrane CENTRAL | (neurofeedback) AND (attention or attention-deficit/hyperactivity disorder or ADHD) | Trials | 2022/06/10 | 354 |
| ScienceDirect | (neurofeedback) AND (attention or attention-deficit/hyperactivity disorder or ADHD) | Research article | 2022/06/10 | 210 |
| ClinicalTrials.gov | (ADHD) AND (neurofeedback) | N/A | 2022/06/10 | 27 |

Abbreviation: N/A: not applied; RCT randomized controlled trials

**Supplemental Table 2. Reasons for study exclusion (n=53)**

| Reason | Number of studies | References |
| --- | --- | --- |
| Not targeting ADHD patients | 9 | [1-9] |
| Not RCT | 10 | [10-19] |
| Duplicated sample source | 10 | [20-29] |
| No available data for analysis | 7 | [30-36] |
| Not EEG neurofeedback | 8 | [37-44] |
| No combination approach | 9 | [45-53] |

Abbreviations: ADHD: attention deficit and hyperactivity disorders; RCT: randomized controlled trial; EEG neurofeedback: electroencephalographic neurofeedback.

**eTable 3. Sensitivity analyses for meta-analysis using leave-one-out approach**

| Outcomes | Number of studies | Effect sizes (95%CI) | Effect size p value | Heterogeneity I^2^ (%) |
| --- | --- | --- | --- | --- |
| Parents Total score | 5 | 0.2898 (0.0238; 0.5557) | 0.0327* | 28.3% |
| **Leave one study out** |  |  |  |  |
| Li (2013) | 4 | 0.2607 (-0.0712; 0.5925) | 0.1237 | 41.0% |
| Lee (2016) | 4 | 0.2166 (-0.0384; 0.4716) | 0.0960 | 9.2% |
| Tang (2014) | 4 | 0.2501 (-0.0720; 0.5722) | 0.1281 | 38.5% |
| Parents Total score(Follow up ) | 3 | 0.4807 (-0.2430; 1.2044) | 0.1930 | 83.2% |
| **Leave one study out** |  |  |  |  |
| Duric (2012 | 2 | 0.8232 ( 0.4756; 1.1708) | < 0.0001 | 0.0% |
| Parents inattention | 5 | 0.3274 (0.0493; 0.6055) | 0.0210* | 33.1% |
| **Leave one study out** |  |  |  |  |
| Li (2013) | 4 | 0.3292 (-0.0365; 0.6949) | 0.0777 | 49.3% |
| Lee (2016) | 4 | 0.2584 (-0.0200; 0.5368) | 0.0689 | 25.2% |
| Tang (2014) | 4 | 0.2358 (-0.0372; 0.5088) | 0.0904 | 24.9% |
| Parents inattention(Follow up ) | 3 | 0.3274 (0.0493; 0.6055) | 0.0210* | 33.1% |
| **Leave one study out** |  |  |  |  |
| Li (2013) | 2 | 0.3906 (-0.4702; 1.2513) | 0.3738 | 83.0% |
| Wang (2007) | 2 | 0.4394 (-0.5273; 1.4061) | 0.3730 | 85.6% |
| Parents Hyperactive &Impulsive(Follow up) | 5 | 0.1714 (-0.0544; 0.3971) | 0.1368 | 3.1% |
| **Leave one study out** |  |  |  |  |
| Duric (2012 | 4 | 0.6769 ( 0.3304; 1.0233) | 0.0000 | 0.0% |

**eTable 4. Meta-regression of pre-defined variables of interest. (a) Global symptoms, (b) Inattention and (c) Hyperactivity/impulsivity**

**(a) Global symptoms**

| **Covariate** | **No. of studies** | **Meta-regression** | | **Proportion of variance explained** |
| --- | --- | --- | --- | --- |
|  |  | **ß (95% CI)** | **p value** |  |
| Session | 5 | -0.0159(-0.0415 ;0.0098) | 0.2248 | 28.99% |
| Age | 5 | -0.1207(-0.4181;0.1767) | 0.4264 | 0.00% |
| Female proportion | 5 | 0.0116(-0.0108;0.0341) | 0.3106 | 8.72% |
| IQ | 3 | 0.0453(-0.0304;0.1209) | 0.2407 | 6.73% |
| Duration | 4 | -0.0440(-0.0978;0.0098) | 0.1089 | 88.40% |

**(b) Inattention**

| **Covariate** | **No. of studies** | **Meta-regression** | | **Proportion of variance explained** |
| --- | --- | --- | --- | --- |
|  |  | **ß (95% CI)** | **p value** |  |
| Session | 5 | -0.0207(-0.0436;0.0022) . | 0.0760 | 100.00% |
| Age | 5 | -0.1763(-0.4560;0.1033) | 0.2165 | 10.33% |
| Female proportion | 5 | 0.0173(-0.0022;0.0367) | 0.0828 | 99.99% |
| IQ | 3 | 0.0446(-0.0228;0.1120) | 0.1951 | 32.84% |
| Duration | 4 | -0.0494(-0.1116;0.0129) | 0.1203 | 62.08% |

**(c) Hyperactivity/impulsivity**

| **Covariate** | **No. of studies** | **Meta-regression** | | **Proportion of variance explained** |
| --- | --- | --- | --- | --- |
|  |  | **ß (95% CI)** | **p value** |  |
| Session | 5 | -0.0128(-0.0354;0.0099) | 0.2693 | 96.28% |
| Age | 5 | -0.0963(-0.3292;0.1366) | 0.4176 | 77.92% |
| Female proportion | 5 | 0.0064(-0.0127;0.0256) | 0.5094 | 0.00% |
| IQ | 3 | 0.0463(-0.0290;0.1217) | 0.2282 | 14.18% |
| Duration | 4 | -0.0308 (-0.0889;0.0273) | 0.2994 | 0.00% |

PRISMA checklist

| **Section and Topic** | **Item #** | **Checklist item** | **Location where item is reported** |
| --- | --- | --- | --- |
| **TITLE** | | |  |
| Title | 1 | Identify the report as a systematic review. | Page 1 |
| **ABSTRACT** | | |  |
| Abstract | 2 | See the PRISMA 2020 for Abstracts checklist. | Page 3 |
| **INTRODUCTION** | | |  |
| Rationale | 3 | Describe the rationale for the review in the context of existing knowledge. | Page 4-6 |
| Objectives | 4 | Provide an explicit statement of the objective(s) or question(s) the review addresses. | Page 6 |
| **METHODS** | | |  |
| Eligibility criteria | 5 | Specify the inclusion and exclusion criteria for the review and how studies were grouped for the syntheses. | Page 7 |
| Information sources | 6 | Specify all databases, registers, websites, organisations, reference lists and other sources searched or consulted to identify studies. Specify the date when each source was last searched or consulted. | Page 7, eTable 1 |
| Search strategy | 7 | Present the full search strategies for all databases, registers and websites, including any filters and limits used. | Page 7, eTable 1 |
| Selection process | 8 | Specify the methods used to decide whether a study met the inclusion criteria of the review, including how many reviewers screened each record and each report retrieved, whether they worked independently, and if applicable, details of automation tools used in the process. | Page 7-8 |
| Data collection process | 9 | Specify the methods used to collect data from reports, including how many reviewers collected data from each report, whether they worked independently, any processes for obtaining or confirming data from study investigators, and if applicable, details of automation tools used in the process. | Page 7-8 |
| Data items | 10a | List and define all outcomes for which data were sought. Specify whether all results that were compatible with each outcome domain in each study were sought (e.g. for all measures, time points, analyses), and if not, the methods used to decide which results to collect. | Page 8 |
|  | 10b | List and define all other variables for which data were sought (e.g. participant and intervention characteristics, funding sources). Describe any assumptions made about any missing or unclear information. | Page 8 |
| Study risk of bias assessment | 11 | Specify the methods used to assess risk of bias in the included studies, including details of the tool(s) used, how many reviewers assessed each study and whether they worked independently, and if applicable, details of automation tools used in the process. | Page 8 |
| Effect measures | 12 | Specify for each outcome the effect measure(s) (e.g. risk ratio, mean difference) used in the synthesis or presentation of results. | Page 8 |
| Synthesis methods | 13a | Describe the processes used to decide which studies were eligible for each synthesis (e.g. tabulating the study intervention characteristics and comparing against the planned groups for each synthesis (item #5)). | Page 8-10 |
|  | 13b | Describe any methods required to prepare the data for presentation or synthesis, such as handling of missing summary statistics, or data conversions. | Page8-10 |
|  | 13c | Describe any methods used to tabulate or visually display results of individual studies and syntheses. | Page8-10 |
|  | 13d | Describe any methods used to synthesize results and provide a rationale for the choice(s). If meta-analysis was performed, describe the model(s), method(s) to identify the presence and extent of statistical heterogeneity, and software package(s) used. | Page8-10 |
|  | 13e | Describe any methods used to explore possible causes of heterogeneity among study results (e.g. subgroup analysis, meta-regression). | Page9-10 |
|  | 13f | Describe any sensitivity analyses conducted to assess robustness of the synthesized results. | Page9-10 |
| Reporting bias assessment | 14 | Describe any methods used to assess risk of bias due to missing results in a synthesis (arising from reporting biases). | Page8 |
| Certainty assessment | 15 | Describe any methods used to assess certainty (or confidence) in the body of evidence for an outcome. | Page8-10 |
| **RESULTS** | | |  |
| Study selection | 16a | Describe the results of the search and selection process, from the number of records identified in the search to the number of studies included in the review, ideally using a flow diagram. | Page 11, Figure 1 |
|  | 16b | Cite studies that might appear to meet the inclusion criteria, but which were excluded, and explain why they were excluded. | Page 11, eTable 2 |
| Study characteristics | 17 | Cite each included study and present its characteristics. | Page 11, Table 1 |
| Risk of bias in studies | 18 | Present assessments of risk of bias for each included study. | Page 11, Figure 2A and B |
| Results of individual studies | 19 | For all outcomes, present, for each study: (a) summary statistics for each group (where appropriate) and (b) an effect estimate and its precision (e.g. confidence/credible interval), ideally using structured tables or plots. | Page 11-12, Table 2  Figure 3-4, |
| Results of syntheses | 20a | For each synthesis, briefly summarise the characteristics and risk of bias among contributing studies. | Page 11, Figure 2A and B |
|  | 20b | Present results of all statistical syntheses conducted. If meta-analysis was done, present for each the summary estimate and its precision (e.g. confidence/credible interval) and measures of statistical heterogeneity. If comparing groups, describe the direction of the effect. | Page 11-13, Table 2,  Figure 3-4,  eTable 3 |
|  | 20c | Present results of all investigations of possible causes of heterogeneity among study results. | Page 11-13 Table 2  Figure 3-4, |
|  | 20d | Present results of all sensitivity analyses conducted to assess the robustness of the synthesized results. | Page 12-13, eTable 3 |
| Reporting biases | 21 | Present assessments of risk of bias due to missing results (arising from reporting biases) for each synthesis assessed. | N/A |
| Certainty of evidence | 22 | Present assessments of certainty (or confidence) in the body of evidence for each outcome assessed. | N/A |
| **DISCUSSION** | | |  |
| Discussion | 23a | Provide a general interpretation of the results in the context of other evidence. | Page 14-17 |
|  | 23b | Discuss any limitations of the evidence included in the review. | Page 16-17 |
|  | 23c | Discuss any limitations of the review processes used. | Page 16-17 |
|  | 23d | Discuss implications of the results for practice, policy, and future research. | Page 17 |
| **OTHER INFORMATION** | | |  |
| Registration and protocol | 24a | Provide registration information for the review, including register name and registration number, or state that the review was not registered. | Page 7 |
|  | 24b | Indicate where the review protocol can be accessed, or state that a protocol was not prepared. | Page 7 |
|  | 24c | Describe and explain any amendments to information provided at registration or in the protocol. | N/A |
| Support | 25 | Describe sources of financial or non-financial support for the review, and the role of the funders or sponsors in the review. | Page 17 |
| Competing interests | 26 | Declare any competing interests of review authors. | Page 17 |
| Availability of data, code and other materials | 27 | Report which of the following are publicly available and where they can be found: template data collection forms; data extracted from included studies; data used for all analyses; analytic code; any other materials used in the review. | Page 6 |

*From:* Page MJ, McKenzie JE, Bossuyt PM, Boutron I, Hoffmann TC, Mulrow CD, et al. The PRISMA 2020 statement: an updated guideline for reporting systematic reviews. BMJ 2021;372:n71. doi: 10.1136/bmj.n71

**References**

1. Morales-Quezada, L., et al., *Neurofeedback impacts cognition and quality of life in pediatric focal epilepsy: An exploratory randomized double-blinded sham-controlled trial.* Epilepsy Behav, 2019. **101**(Pt A): p. 106570.

2. Bioulac, S., et al., *Personalized at-home neurofeedback compared with long-acting methylphenidate in an european non-inferiority randomized trial in children with ADHD.* BMC Psychiatry, 2019. **19**(1): p. 237.

3. Jirayucharoensak, S., et al., *A game-based neurofeedback training system to enhance cognitive performance in healthy elderly subjects and in patients with amnestic mild cognitive impairment.* Clin Interv Aging, 2019. **14**: p. 347-360.

4. Ratcliff, C.G., et al., *A Randomized Controlled Trial of Brief Mindfulness Meditation for Women Undergoing Stereotactic Breast Biopsy.* J Am Coll Radiol, 2019. **16**(5): p. 691-699.

5. Yeo, S.N., et al., *Effectiveness of a Personalized Brain-Computer Interface System for Cognitive Training in Healthy Elderly: A Randomized Controlled Trial.* J Alzheimers Dis, 2018. **66**(1): p. 127-138.

6. Azizi, A., F.M. Drikvand, and M.A. Sepahvandi, *Comparison of the Effect of Cognitive Rehabilitation and Neurofeedback on Sustained Attention Among Elementary School Students with Specific Learning Disorder: A Preliminary Randomized Controlled Clinical Trial.* Appl Psychophysiol Biofeedback, 2018. **43**(4): p. 301-307.

7. Rostami, R., et al., *Effects of neurofeedback on the short-term memory and continuous attention of patients with moderate traumatic brain injury: A preliminary randomized controlled clinical trial.* Chin J Traumatol, 2017. **20**(5): p. 278-282.

8. Schabus, M., et al., *Better than sham? A double-blind placebo-controlled neurofeedback study in primary insomnia.* Brain, 2017. **140**(4): p. 1041-1052.

9. Keith, J.R., et al., *An assessment of an automated EEG biofeedback system for attention deficits in a substance use disorders residential treatment setting.* Psychol Addict Behav, 2015. **29**(1): p. 17-25.

10. Holtmann, M., et al., *Neurofeedback in children with attention-deficit/hyperactivity disorder (ADHD)--a controlled multicenter study of a non-pharmacological treatment approach.* BMC Pediatr, 2014. **14**: p. 202.

11. Riesco-Matías, P., et al., *What Do Meta-Analyses Have to Say About the Efficacy of Neurofeedback Applied to Children With ADHD? Review of Previous Meta-Analyses and a New Meta-Analysis.* J Atten Disord, 2021. **25**(4): p. 473-485.

12. Van Doren, J., et al., *Sustained effects of neurofeedback in ADHD: a systematic review and meta-analysis.* Eur Child Adolesc Psychiatry, 2019. **28**(3): p. 293-305.

13. Rossiter, T., *The effectiveness of neurofeedback and stimulant drugs in treating AD/HD: part II. Replication.* Appl Psychophysiol Biofeedback, 2004. **29**(4): p. 233-43.

14. Fuchs, T., et al., *Neurofeedback treatment for attention-deficit/hyperactivity disorder in children: a comparison with methylphenidate.* Appl Psychophysiol Biofeedback, 2003. **28**(1): p. 1-12.

15. Ryoo, M. and C. Son, *Effects of Neurofeekback Training on EEG, Continuous Performance Task (CPT), and ADHD Symptoms in ADHD-prone College Students.* J Korean Acad Nurs, 2015. **45**(6): p. 928-38.

16. Qian, X., et al., *Brain-computer-interface-based intervention re-normalizes brain functional network topology in children with attention deficit/hyperactivity disorder.* Transl Psychiatry, 2018. **8**(1): p. 149.

17. Pakdaman, F., et al., *The efficacy of Ritalin in ADHD children under neurofeedback training.* Neurol Sci, 2018. **39**(12): p. 2071-2078.

18. Coben, R., D.C. Hammond, and M. Arns, *19 Channel Z-Score and LORETA Neurofeedback: Does the Evidence Support the Hype?* Appl Psychophysiol Biofeedback, 2019. **44**(1): p. 1-8.

19. Heinrich, H., et al., *Effects of neurofeedback on the dysregulation profile in children with ADHD: SCP NF meets SDQ-DP - a retrospective analysis.* Psychol Med, 2020. **50**(2): p. 258-263.

20. Gevensleben, H., et al., *Distinct EEG effects related to neurofeedback training in children with ADHD: a randomized controlled trial.* Int J Psychophysiol, 2009. **74**(2): p. 149-57.

21. Janssen, T.W., et al., *A randomized controlled trial into the effects of neurofeedback, methylphenidate, and physical activity on EEG power spectra in children with ADHD.* J Child Psychol Psychiatry, 2016. **57**(5): p. 633-44.

22. Meisel, V., et al., *Neurofeedback and standard pharmacological intervention in ADHD: a randomized controlled trial with six-month follow-up.* Biol Psychol, 2013. **94**(1): p. 12-21.

23. Baumeister, S., et al., *The impact of successful learning of self-regulation on reward processing in children with ADHD using fMRI.* Atten Defic Hyperact Disord, 2019. **11**(1): p. 31-45.

24. Beauregard, M. and J. Lévesque, *Functional magnetic resonance imaging investigation of the effects of neurofeedback training on the neural bases of selective attention and response inhibition in children with attention-deficit/hyperactivity disorder.* Appl Psychophysiol Biofeedback, 2006. **31**(1): p. 3-20.

25. Aggensteiner, P.M., et al., *Slow cortical potentials neurofeedback in children with ADHD: comorbidity, self-regulation and clinical outcomes 6 months after treatment in a multicenter randomized controlled trial.* Eur Child Adolesc Psychiatry, 2019. **28**(8): p. 1087-1095.

26. Bink, M., et al., *1-year follow-up of neurofeedback treatment in adolescents with attention-deficit hyperactivity disorder: randomised controlled trial.* BJPsych Open, 2016. **2**(2): p. 107-115.

27. Steiner, N.J., et al., *In-school neurofeedback training for ADHD: sustained improvements from a randomized control trial.* Pediatrics, 2014. **133**(3): p. 483-92.

28. Vollebregt, M.A., et al., *Does EEG-neurofeedback improve neurocognitive functioning in children with attention-deficit/hyperactivity disorder? A systematic review and a double-blind placebo-controlled study.* J Child Psychol Psychiatry, 2014. **55**(5): p. 460-72.

29. Liechti, M.D., et al., *First clinical trial of tomographic neurofeedback in attention-deficit/hyperactivity disorder: evaluation of voluntary cortical control.* Clin Neurophysiol, 2012. **123**(10): p. 1989-2005.

30. Norouzi, E., F. Hossieni, and M. Solymani, *Effects of Neurofeedback Training on Performing Bimanual Coordination In-phase and Anti-phase Patterns in Children with ADHD.* Appl Psychophysiol Biofeedback, 2018. **43**(4): p. 283-292.

31. Logemann, H.N., et al., *The effectiveness of EEG-feedback on attention, impulsivity and EEG: a sham feedback controlled study.* Neurosci Lett, 2010. **479**(1): p. 49-53.

32. Baumeister, S., et al., *Neurofeedback Training Effects on Inhibitory Brain Activation in ADHD: A Matter of Learning?* Neuroscience, 2018. **378**: p. 89-99.

33. Döpfner, M., et al., *ESCAschool study: trial protocol of an adaptive treatment approach for school-age children with ADHD including two randomised trials.* BMC Psychiatry, 2017. **17**(1): p. 269.

34. Mohagheghi, A., et al., *A Randomized Trial of Comparing the Efficacy of Two Neurofeedback Protocols for Treatment of Clinical and Cognitive Symptoms of ADHD: Theta Suppression/Beta Enhancement and Theta Suppression/Alpha Enhancement.* Biomed Res Int, 2017. **2017**: p. 3513281.

35. Thompson, L. and M. Thompson, *Neurofeedback combined with training in metacognitive strategies: effectiveness in students with ADD.* Appl Psychophysiol Biofeedback, 1998. **23**(4): p. 243-63.

36. Schönenberg, M., et al., *Neurofeedback, sham neurofeedback, and cognitive-behavioural group therapy in adults with attention-deficit hyperactivity disorder: a triple-blind, randomised, controlled trial.* Lancet Psychiatry, 2017. **4**(9): p. 673-684.

37. Rubia, K., et al., *Functional connectivity changes associated with fMRI neurofeedback of right inferior frontal cortex in adolescents with ADHD.* Neuroimage, 2019. **188**: p. 43-58.

38. Zilverstand, A., et al., *fMRI Neurofeedback Training for Increasing Anterior Cingulate Cortex Activation in Adult Attention Deficit Hyperactivity Disorder. An Exploratory Randomized, Single-Blinded Study.* Plos One, 2017. **12**(1): p. e0170795.

39. Criaud, M., et al., *Increased left inferior fronto-striatal activation during error monitoring after fMRI neurofeedback of right inferior frontal cortex in adolescents with attention deficit hyperactivity disorder.* Neuroimage Clin, 2020. **27**: p. 102311.

40. Sherwood, M.S., et al., *Self-directed down-regulation of auditory cortex activity mediated by real-time fMRI neurofeedback augments attentional processes, resting cerebral perfusion, and auditory activation.* Neuroimage, 2019. **195**: p. 475-489.

41. Lam, S.L., et al., *Neurofunctional and behavioural measures associated with fMRI-neurofeedback learning in adolescents with Attention-Deficit/Hyperactivity Disorder.* Neuroimage Clin, 2020. **27**: p. 102291.

42. Takamura, M., et al., *Antidepressive effect of left dorsolateral prefrontal cortex neurofeedback in patients with major depressive disorder: A preliminary report.* J Affect Disord, 2020. **271**: p. 224-227.

43. Kim, H.C., et al., *Mediation analysis of triple networks revealed functional feature of mindfulness from real-time fMRI neurofeedback.* Neuroimage, 2019. **195**: p. 409-432.

44. Lee, G.J. and J.A. Suhr, *Expectancy Effects on Self-Reported Attention-Deficit/Hyperactivity Disorder Symptoms in Simulated Neurofeedback: A Pilot Study.* Arch Clin Neuropsychol, 2019. **34**(2): p. 200-205.

45. Sudnawa, K.K., et al., *Effectiveness of neurofeedback versus medication for attention-deficit/hyperactivity disorder.* Pediatr Int, 2018. **60**(9): p. 828-834.

46. Meisel, V., et al., *Reprint of "Neurofeedback and standard pharmacological intervention in ADHD: a randomized controlled trial with six-month follow-up".* Biol Psychol, 2014. **95**: p. 116-25.

47. Ogrim, G. and K.A. Hestad, *Effects of neurofeedback versus stimulant medication in attention-deficit/hyperactivity disorder: a randomized pilot study.* J Child Adolesc Psychopharmacol, 2013. **23**(7): p. 448-57.

48. Lévesque, J., M. Beauregard, and B. Mensour, *Effect of neurofeedback training on the neural substrates of selective attention in children with attention-deficit/hyperactivity disorder: a functional magnetic resonance imaging study.* Neurosci Lett, 2006. **394**(3): p. 216-21.

49. Steiner, N.J., et al., *Computer-based attention training in the schools for children with attention deficit/hyperactivity disorder: a preliminary trial.* Clin Pediatr (Phila), 2011. **50**(7): p. 615-22.

50. Barth, B., et al., *A randomized-controlled neurofeedback trial in adult attention-deficit/hyperactivity disorder.* Sci Rep, 2021. **11**(1): p. 16873.

51. Lim, C.G., et al., *A randomized controlled trial of a brain-computer interface based attention training program for ADHD.* PLoS One, 2019. **14**(5): p. e0216225.

52. Linden, M., T. Habib, and V. Radojevic, *A controlled study of the effects of EEG biofeedback on cognition and behavior of children with attention deficit disorder and learning disabilities.* Biofeedback Self Regul, 1996. **21**(1): p. 35-49.

53. Bink, M., et al., *Behavioral effects of neurofeedback in adolescents with ADHD: a randomized controlled trial.* Eur Child Adolesc Psychiatry, 2015. **24**(9): p. 1035-48.
